# Unveiling the relation between swallowing muscle mass and skeletal muscle mass in head and neck cancer patients

**DOI:** 10.1007/s00405-025-09207-0

**Published:** 2025-01-25

**Authors:** Javier Hurtado-Oliva, Lucy Núñez-Miranda, Aniek T. Zwart, Jeroen Vister, Boudewijn E. C. Plaat, Roel J. H. M. Steenbakkers, Anouk van der Hoorn, Inge Wegner, Gyorgy B. Halmos

**Affiliations:** 1 Department of Otorhinolaryngology, Head and Neck Surgery, University Medical Center Groningen, University of Groningen, Hanzeplein 1, PO box 30.001, Groningen, 9700RB The Netherlands; 2 Departamento de Fonoaudiología, Facultad de Medicina, Universidad de Chile, Santiago, Chile; 3 Departamento de Procesos Terapéuticos, Facultad de Ciencias de la Salud, Universidad Católica de Temuco, Temuco, Chile; 4 Department of Radiation Oncology, University Medical Center Groningen, University of Groningen, Groningen, The Netherlands; 5 Department of Radiology, University Medical Center Groningen, University of Groningen, Groningen, The Netherlands

**Keywords:** Head and neck cancer, Swallowing muscle mass, Skeletal muscle mass, Sarcopenia, Dysphagia

## Abstract

**Purpose:**

Sarcopenia, characterized by loss of skeletal muscle mass (SMM) and strength, often leads to dysphagia in the elderly. This condition can also worsen treatment outcomes in head and neck cancer (HNC) patients, who are susceptible to swallowing difficulties. This study aimed to establish the correlation between swallowing muscle mass (SwMM) and SMM in HNC patients.

**Methods:**

Data from 157 HNC patients in the OncoLifeS biobank of the University Medical Center Groningen were analyzed using pre-treatment neck CT scans. The SwMM was assessed by the cross-sectional area (CSA) of the tongue complex muscles (TCM), and SMM was indicated by the skeletal muscle index (SMI), calculated from corrected CSA at the third lumbar vertebra (L3). Correlations between SwMM and SMM were analyzed using Pearson or Spearman tests, and multivariable linear regression with SMI as dependent variable was performed.

**Results:**

SwMM was moderately correlated with SMI (*r* = 0.600, *p* < 0.001), CSA at C3 (*r* = 0.538, *p* < 0.001), and CSA at L3 (*r* = 0.651, *p* < 0.001). The CSA at C3 strongly correlated with SMI (*r* = 0.871, *p* < 0.001). In multivariable regression analysis, age, sex, and weight were strong predictors of SMI, while the TCM area was a less robust predictor (*p* = 0.059). Models with CSA at C3 and L3 showed all variables as significant predictors (*p* < 0.001).

**Conclusions:**

Although SwMM was significantly correlated with SMI and holds clinical utility, it is not strong enough to be considered interchangeably with C3 for predicting SMI, suggesting that swallowing muscles represent a different entity than skeletal muscles and not reflect accurately the general muscle mass.

**Supplementary Information:**

The online version contains supplementary material available at 10.1007/s00405-025-09207-0.

## Introduction

Sarcopenia is a well-recognized geriatric syndrome, defined as a generalized skeletal muscle disorder characterized by reduced muscle volume and strength, and consequently decreased physical performance [1]. Its prevalence increases with age and is often exacerbated by malnutrition, making it particularly notable in patients with head and neck cancer (HNC) who suffer from tumor-induced dysphagia [2]. Sarcopenia impairs HNC treatment outcome, reflected by prolonged treatment, treatment interruptions, increased toxicity, surgical complications, compromised quality of life, and reduced survival rates [3, 4].

Various methods and tools are available to assess muscle strength, muscle quantity, and physical performance for diagnosing sarcopenia [1, 5, 6]. Computed tomography (CT) imaging of a single slice at the level of the third lumbar vertebra (L3) enables the calculation of the skeletal muscle index (SMI), which is currently considered the gold standard for quantifying skeletal muscle mass (SMM) due to its strong correlation with total body muscle mass and thereby facilitating the diagnosis of radiological-defined sarcopenia [7–11]. Since abdominal CT scans are uncommon in HNC patients, Swartz et al. developed a method to assess SMM using a single CT slice at the third cervical vertebra (C3), typically included in standard head and neck scans [12]. With all these advances, a low skeletal muscle mass (SMM) has emerged as a significant biomarker for predicting adverse events during treatment and worse survival in HNC, underscoring its evolving importance in clinical practice [7, 13].

Sarcopenia also adversely affects swallowing safety and efficiency due to declining swallowing muscle mass (SwMM) function [14]. This condition could impact dietary intake and quality of life, and increase the risks of aspiration pneumonia, malnutrition, dehydration, morbidity, and mortality [15–17]. In HNC patients, it could further exacerbate these complications and worsen cancer treatment outcomes [18, 19].

Despite the recognized impact of sarcopenia on SMM, its specific effects on SwMM remain unclear, acknowledging a complex relationship and overlap between sarcopenia, malnutrition, frailty, and dysphagia [17, 20]. Given the critical roles of these different muscle groups, investigating their relationship could provide insights to improve patient management and outcomes.

Therefore, this study aims to explore whether the swallowing muscle mass is correlated with the skeletal muscle mass in HNC patients.

## Materials and methods

### Ethical considerations

In this study, data were retrospectively collected from the Oncological Life Study (OncoLifeS), a large prospective oncological data-biobank approved by the Medical Ethical Committee of the University Medical Center Groningen (UMCG) (Supplemental reference, S1). The OncoLifeS database is registered in the Netherlands Trial Register under reference number NL7839. The study protocol was approved by the scientific board of OncoLifeS. All patients provided written informed consent, and all data and used CT scans were pseudonymized in compliance with data protection regulations.

### Study population

HNC patients were reselected from a previous study [21]. All patients diagnosed with a primary mucosal malignancy located at the lip and oral cavity, oropharynx, hypopharynx, or larynx, of any age, regardless of the stage of disease, and treatment modality were included. Patients were diagnosed at the Department of Otorhinolaryngology and Head and Neck Surgery, and at the Department of Oral and Maxillofacial Surgery of the UMCG between October 2014 and June 2020. All patients underwent a pre-treatment neck CT scan, as part of the standard oncological work-up. Cases were excluded in case of tumors located in the nasal cavity/paranasal sinus, only a magnetic resonance imaging (MRI) scan was performed, significant oblique position of the head and neck, significant motion / streak artifacts in the CT scan, poor imaging quality, abnormal tongue anatomy (i.e., severe asymmetry, deformities because of tumor) or tumor infiltration at the level of the SwMM measurements.

Demographic and clinical baseline data retrieved from the OncoLifeS database included age (years), sex, weight (kg), height (m), body mass index (BMI, kg/m^2^), localization of the tumor, and stage of the disease. Tumor staging was performed according to the Union for International Cancer Control, TNM Classification of Malignant Tumors 7th edition (S2), as TNM7 was operational at the time of inclusion.

### Measurements of muscle mass

Muscle analysis was performed using the Aquarius workstation iNtuition Edition (ver.4.7.0.22–111) program on post-contrast head and neck CT scans reconstructed with a 1.0–2.0 mm slice thickness, and a soft-tissue kernel.

**Fig. 1:**
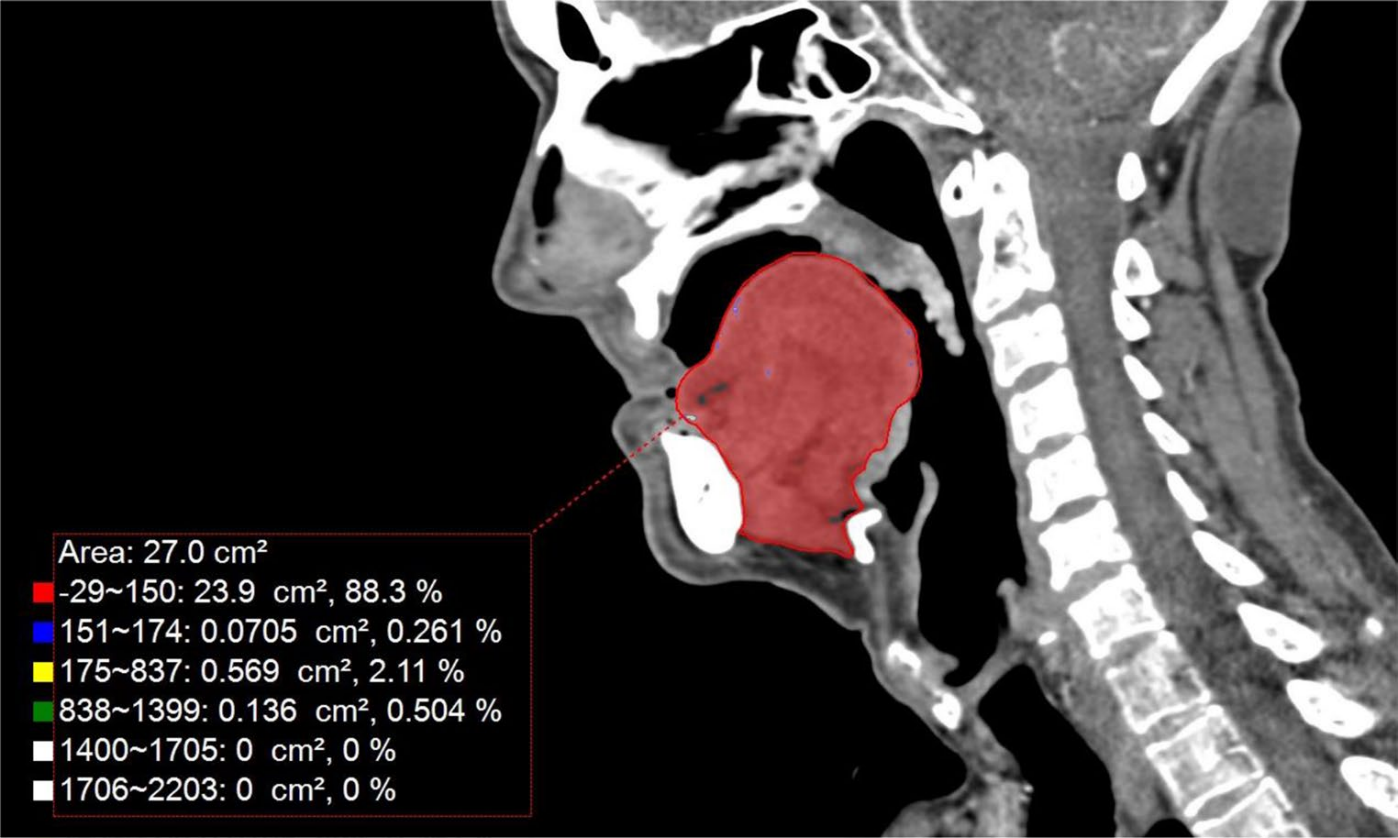
Example of the tongue complex muscles area measurement on a mid-sagittal CT slice. The red area represents the delineated muscles. The upper value (area: 27.0 cm^2^) corresponds to the sagittal area of the tongue complex muscle. Other values with color squares correspond to measurements with Hounsfield unit threshold tool for different muscle densities. Note, that lymphoid tissue was excluded from the delineation at the base of the tongue

The SwMM was measured using mid-sagittal CT scans at the head and neck level, following a previously established method [22]. The total SwMM volume was defined as the summed volume of the pharyngeal constrictor muscles, genioglossus muscle, and the mylohyoid/geniohyoid complex muscle. The delineation and measurement of the mid-sagittal TCM area included the genioglossus muscle, mylohyoid/geniohyoid muscles, and the intrinsic tongue musculature, excluding lymphoid tissue at the tongue base. Because in the above mentioned earlier study [28], the sagittal area of the tongue complex muscles (TCM, cm^2^) had a strong, positive and statistically significant correlation with the total SwMM volume, in this study, TCM area was defined as an indicator for SwMM. All TCM measurements were performed by two researchers, and cases were supervised by one head and neck surgeon. An experienced head and neck radiologist was consulted in selected cases, when identification of muscle borders was hard. See Fig. 1 for TCM area measurements.

The SMM was measured at the levels of the C3 and L3 using CT scans, following previous validated methods [12]. For both, the CSA was defined as the summed area of skeletal muscle using a standard Hounsfield Unit (HU) threshold in a range of -29 to + 150 HU [8]. At C3, the cross-sectional area (CSA, cm^2^) of SMM was measured by delineating the right and left sternocleidomastoid muscles and the paravertebral muscles as described earlier [12]. The CSA of SMM at L3 was calculated according to Eq. 1 (12). The CSA at L3 was then adjusted for the patient’s height (m^2^) to yield the SMI, as shown in Eq. 2 (11). Therefore, SMI was defined as an indicator for SMM, calculated with the estimated CSA at L3, using CSA at C3.

1 $$\eqalign{& CSA{\rm{ }}of{\rm{ }}SMM{\rm{ }}at{\rm{ }}L3{\rm{ }}\left( {c{m^2}} \right){\rm{ }} = {\rm{ }}27.304{\rm{ }} + {\rm{ }}1.363 \cr & *total{\rm{ }}CSA{\rm{ }}of{\rm{ }}SMM{\rm{ }}at{\rm{ }}C3{\rm{ }}\left( {c{m^2}} \right){\rm{ }} \cr & - {\rm{ }}0.671*Age{\rm{ }}\left( {years} \right){\rm{ }} \cr & + {\rm{ }}0.640*Weight{\rm{ }}\left( {kg} \right){\rm{ }} \cr & + {\rm{ }}26.442*Sex{\rm{ }}\left( {1{\rm{ }}for{\rm{ }}female,{\rm{ }}and{\rm{ }}2{\rm{ }}for{\rm{ }}male} \right) \cr} $$

2 $$SMI{\rm{ }}\left( {c{m^2}/{\rm{ }}{m^2}} \right){\rm{ }} = {\rm{ }}CSA{\rm{ }}at{\rm{ }}L3{\rm{ }}\left( {c{m^2}} \right){\rm{ }}/{\rm{ }}Height{\rm{ }}\left( {{m^2}} \right)$$

### Statistical analyses

Descriptive statistics for patient and clinical baseline data were presented as frequencies with percentages, or as mean scores with their standard deviations (SDs). The normality distribution of TCM and SMM measurements was assessed using the Shapiro-Wilk test and visual inspections of Q-Q plots. Pearson correlation coefficients were calculated for TCM area, SMM at L3 level, and SMI because of normal distributed data, while Spearman correlation coefficients were used for SMM measurements at the C3 level because of not-normal distributed data. Differences between age groups (*≥* 65 years versus < 65 years) and sex were analyzed through independent samples t-tests. A multivariable linear regression analysis was conducted to investigate the impact of TCM CSA, CSA at C3, and CSA at L3 on SMI as dependent variable, including age, sex, and weight as demographic predictor variables to each model, in concordance with previous research of Swartz et al. [12]. This analysis followed the predictive model framework outlined by Swartz et al. [13]. Coefficients (β) and *p*-values (threshold of 0.1) were analyzed to determine the significance and direction of the relationships between each predictor and the SMI. Adjusted R² value was considered to assess the goodness of fit of each model. Statistical analyses were conducted using Statistical Package for the Social Sciences (SPSS) statistics software version 28.0 (IBM Corp., Armonk, New York).

## Results

### Patient characteristics

**Fig. 2:**
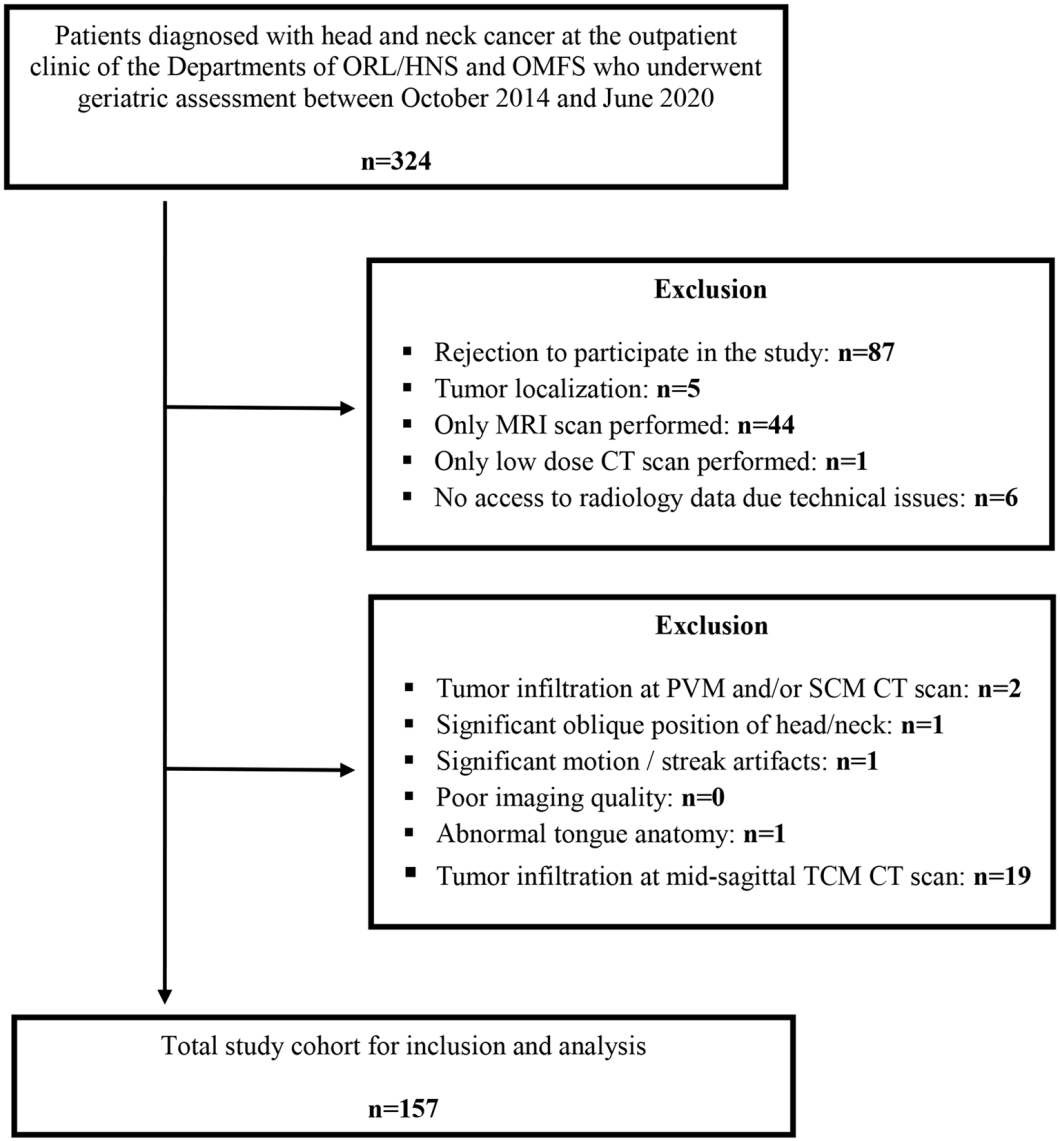
Flowchart diagram of patient cohort for analysis Legend: ORL/HNS = otorhinolaryngology/head and neck surgery; OMFS = oral and maxillofacial surgery; MRI = Magnetic Resonance Imaging; PVM = paravertebral muscles; SCM = sternocleidomastoid muscles; TCM = tongue complex muscles

In this study, a total of 324 patients were initially included, with 157 remaining eligible for analyses after applying the exclusion criteria (Fig. 2). The mean age of the final study population was 65.7 years (SD 9.5), with the majority being over 65 years old (57.3%). Most patients were male (76.4%). The average weight was 78.9 kg (SD 17.8), and the mean BMI was 25.6 kg/m² (SD 5.1). The most prevalent tumor localizations were the larynx (39.5%) and the oropharynx (35.0%), with the majority of cases diagnosed at an advanced stage, specifically stage IV (59.9%). Squamous cell carcinoma was the predominant histological type (96.2%) (Table 1).

**Table 1:** Demographic and clinical characteristics

Variable	*N* (%)
Age (years, mean, SD) * ≥*65 years <65 years	65.7 (*±* 9.5) 90 (57.3) 67 (42.7)
Sex Male Female	120 (76.4) 37 (23.6)
Weight (kg, mean, SD)	78.9 (*±* 17.8)
BMI (kg/m^2^, mean, SD)	25.6 (*±* 5.1)
Tumor localization Larynx Oropharynx Lip and oral cavity Hypopharynx	62 (39.5) 55 (35.0) 26 (16.6) 14 (8.9)
Tumor Stage I II III IV	10 (6.4) 19 (12.1) 34 (21.7) 94 (59.9)
Histopathology Squamous Cell Carcinoma Other	151 (96.2) 6 (3.8)

Legend: BMI = body mass index; SD = standard deviation

### Radiological findings

#### Muscle mass measurements

In the SwMM analysis, the mean area of the TCM was 29.5 cm² (SD 4.4). For the SMM, the CSA at the C3 vertebra was 41.9 cm² (SD 9.6), and at the L3 vertebra was 137.5 cm² (SD 29.2). The SMI had an average value of 44.4 cm²/m² (SD 7.9). Regarding muscle mass measurements across sex and age groups, males consistently had significantly higher muscle mass measurements than females across all variables. Conversely, comparisons between age groups (*≥* 65 years vs. < 65 years) showed no significant differences for all variables (Table 2).

**Table 2:** Muscle mass area and skeletal muscle mass index measurements

Variable	Category	Mean (SD)	Mean difference	Independent Samples t-test	
				t-value	p-value
TCM (cm^2^)	Male Female	30.55 (4.04) 26.08 (3.96)	4.46	5.899	**< 0.001**
	*≥* 65 years < 65 years	29.48 (4.16) 29.51 (4.80)	0.02	0.037	0.970
CSA at C3 (cm^2^)	Male Female	45.25 (8.24) 31.23 (4.79)	14.02	12.861	**< 0.001**
	*≥* 65 years < 65 years	41.80 (8.35) 42.14 (11.19)	0.33	0.209	0.835
CSA at L3 (cm^2^)	Male Female	149.50 (20.95) 98.63 (14.71)	50.87	16.496	**< 0.001**
	*≥* 65 years < 65 years	133.98 (26.82) 142.27 (31.75)	8.29	1.770	0.079
SMI (cm^2^/ m^2^)	Male Female	47.29 (6.11) 35.06 (5.53)	12.23	10.872	**< 0.001**
	*≥* 65 years < 65 years	43.50 (7.43) 45.63 (8.42)	2.13	1.680	0.095

Legend: TCM = tongue complex muscles; CSA = cross sectional area; C3 = third cervical vertebrae; L3 = third lumbar vertebrae; SMI = skeletal muscle index. Significant p-values values are indicated in bold

#### The association between swallowing muscle mass, skeletal muscle mass, and skeletal muscle mass index

**Fig. 3:**
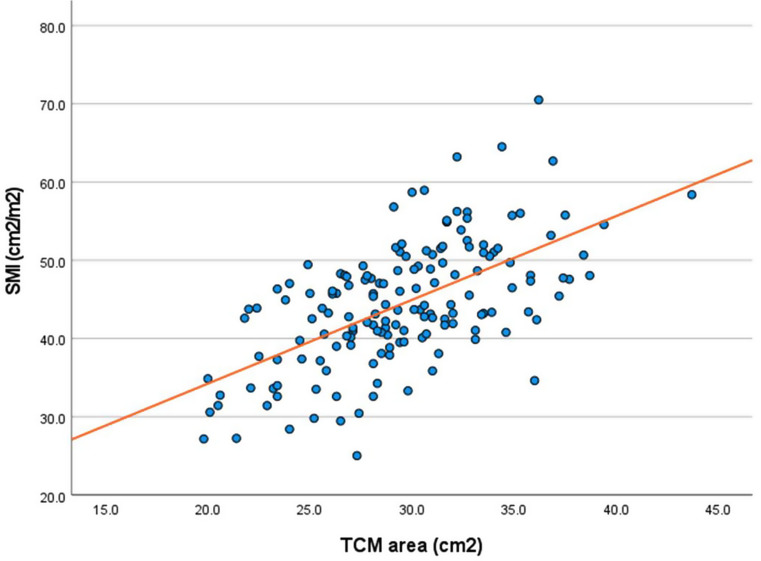
Scatter plot correlation between tongue complex muscle (TCM) area and skeletal muscle index (SMI)

The TCM area had a significant positive correlation with SMI (*r* = 0.600, *p* < 0.001) (Fig. 3). Similarly, significant correlations were observed between TCM area and CSA at C3 (*r* = 0.538, *p* < 0.001), and CSA at L3 (*r* = 0.651, *p* < 0.001). Comparatively, CSA at C3 demonstrated a strong correlation with CSA at L3 (*r* = 0.909, *p* < 0.001), and with SMI (*r* = 0.871, *p* < 0.001). Furthermore, CSA at L3 had a very strong correlation with SMI (*r* = 0.915, *p* < 0.001) (Table 3).

**Table 3:** Correlation coefficient between swallowing muscle mass area and skeletal muscle mass area and index

Correlation analysis	Muscles	*R*	*p*-value
Pearson correlation	TCM - CSA at L3	0.651	**< 0.001**
	TCM - SMI	0.600	**< 0.001**
	CSA at L3 - SMI	0.915	**< 0.001**
Spearman correlation	CSA at C3– TCM	0.538	**< 0.001**
	CSA at C3 - CSA at L3	0.909	**< 0.001**
	CSA at C3 - SMI	0.871	**< 0.001**

Legend: TCM = tongue complex muscles; CSA = cross sectional area; C3 = third cervical vertebrae; L3 = third lumbar vertebrae; SMI = skeletal muscle index. Significant p-values values are indicated in bold

The multivariable regression analysis, based on the predictive model framework by Swartz et al. [12], identified significant predictors among muscle mass measurements and demographic covariates such as age, sex and weight. In the TCM area model, age, sex, and weight were significant strong predictors of SMI (*p* < 0.001), with the TCM area being also a significant predictor of SMI (*p* = 0.059), although less robust compared to the demographic factors. In contrast, the model of CSA at C3 showed that all variables were significant predictors (*p* < 0.001). For the model of CSA at L3, all variables were significant predictors (*p* < 0.001). The regression coefficient for CSA at C3 was the highest (*β* = 0.438), in comparison to CSA at L3 (*β* = 0.322), and TCM (*β* = 0.199). The models of CSA at C3 and CSA at L3, had higher adjusted R^2^ values, in comparison to TCM area model (Table 4).

**Table 4:** Multivariable linear regression analysis

Model	β coefficient (Std. Err.)	*p*-value	Adjusted *R*^2^
TCM area Age Sex Weight	0.199 (0.105) -0.174 (0.036) -9.631 (0.883) 0.201 (0.024)	**0.059** **< 0.001** **< 0.001** **< 0.001**	0.714
CSA at C3 Age Sex Weight	0.438 (0.039) -0.143 (0.027) -4.999 (0.770) 0.131 (0.017)	**< 0.001** **< 0.001** **< 0.001** **< 0.001**	0.840
CSA at L3 Age Sex Weight	0.322 (0.029) 0.073 (0.035) 3.516 (1.366) -0.075 (0.031)	**< 0.001** **0.036** **0.011** **0.016**	0.840

Legend: TCM = tongue complex muscles; CSA = cross sectional area; C3 = third cervical vertebrae; L3 = third lumbar vertebrae; SMI = skeletal muscle index. Significant p-values values are indicated in bold

Dependent variable: SMI

## Discussion

This is the first study investigating the relationship between the SwMM and SMM in HNC patients. Our findings demonstrate statistically significant positive correlations between SwMM and SMM across different vertebra levels, as well as with the SMI, along with demographical factors like age, sex, and weight.

The comparison between SwMM and SMM in this study revealed differences in their predictive values and clinical implications. The skeletal muscle measurements, particularly the CSA at the C3 and L3 vertebra, showed stronger correlations with the SMI compared to the SwMM. This suggests that CSA measurements at C3 and L3 levels may provide a more robust and comprehensive assessment of overall muscle mass. This finding emphasizes the critical role these skeletal muscle groups play in body functions and physical performance, which are key indicators of sarcopenia.

Although SwMM was significantly correlated with SMI and holds clinical utility, it is not strong enough to be considered interchangeably with C3 for predicting SMI. This suggests that swallowing muscles might not reflect the general muscle health as accurately as skeletal muscles and may be specifically true for HNC patients as their swallowing function is often altered directly or indirectly by the tumor. The variability in SwMM and SMM may reflect differing levels of muscle conditioning; some patients may have “well-developed” or “well-trained” swallowing muscles despite overall sarcopenia, or conversely, generalized muscle mass with relatively weaker SwMM. We do not have enough knowledge at this moment to draw firm conclusions.

Several studies have highlighted the relevance of evaluating SwMM to understand its relationship with sarcopenia, swallowing function and related complications. For example, correlations have been demonstrated between the geniohyoid muscle and the tongue with the trunk muscle mass [23], as well as with the SMI [24] in community-dwelling older adults, emphasizing the interconnected nature of muscle groups. Another study found a correlation between SwMM and SMI, involving the total CSA of the geniohyoid, digastric, pharyngeal constrictor, masseter, and temporal muscles, assessed through CT scans in stroke patients [25]. Thus, the study by Pinho et al. provides valuable insights into the physiopathology of the interconnected muscles and aligns with our findings. However, our study uniquely focused on HNC patients, provided a more specific and accurate assessment and definition of SwMM [22], and followed a validated methodology for predictive modeling analysis taking into account demographical factors [12].

Sarcopenia is frequently associated with decreased tongue muscle mass and quality [26], and is a significant predictor of aspiration pneumonia in various populations, including elderly [27] and, HNC patients undergoing chemoradiotherapy [28]. Higher rates of aspiration pneumonia have also been observed in sarcopenic cancer patients undergoing esophagectomy (S3), surgery for colorectal cancer (S4), endoscopic submucosal dissection (S5), and those with alcoholic hepatitis (S6). However, comparing these findings with our study is difficult due to the differences in patient groups. This variability underscores the need for tailored clinical approaches when managing sarcopenia in diverse patient populations.

The association between sarcopenia and dysphagia [29] further supports the importance of a comprehensive muscle assessment to manage sarcopenic dysphagia effectively. In HNC patients, the relationship between SMM and swallowing function has been explored, with findings showing that sarcopenic patients had higher rates of dysphagia compared to non-sarcopenic patients before treatment [30]. This highlights the importance of early intervention and continuous monitoring of muscle health in HNC patients to mitigate dysphagia risk. Several studies have further demonstrated that the CSA of the geniohyoid and tongue muscles is significantly associated with swallowing muscle strength and aspiration risk [31, 32]. Additional correlations have also been found between masticatory muscle mass and SMI at the L3 level [33], between the masseter muscle and CSA at both C3 and L3 levels [34], and between the loss of appendicular SMM and swallowing function [35]. These findings reinforce the impact of skeletal muscle groups on swallowing muscles.

Our findings can be clinically important. Identifying a significant correlation between SwMM and SMM, our study underscores the relation between these muscles with very different functions, potentially allowing for early detection of sarcopenic dysphagia. For HNC patients, understanding the relationship between muscle atrophy and swallowing function may help for a dysphagia risk stratification, contributing to an early intervention of the complex coexistence and relationship between sarcopenia, dysphagia, frailty, malnutrition, and comorbidities [36–38]. For instance, patients with low SMM and SwMM could be identified as at higher risk for dysphagia, enabling proactive management and tailored therapeutic interventions. Integrating SwMM measurement as a standard pre-treatment assessment could provide valuable information for clinical management and to optimize patient outcomes. However, our study involved the TCM area measurements as it correlates with the SwMM, which we defined as the total sum of the pharyngeal constrictor muscles, genioglossus muscle, and the mylohyoid / geniohyoid complex muscles volume, and not a specific muscle correlation. Therefore, a global or muscle-specific intervention tailored program should be carefully developed.

Age-related changes in muscle mass further highlight the importance of considering aging in muscle assessment. Studies have shown that the quantity and quality of swallowing-related muscles deteriorate with aging [39, 40]. The CSA of muscles such as the masseter, genioglossus, geniohyoid, and digastric decreases in older individuals with dysphagia [41]. In our study, age was a significant factor that enhanced the predictive value of the SwMM. In our cohort, no significant difference was observed between age groups using 65 years as a threshold. This finding emphasizes the importance of considering biological age over chronological age, as individual variations in biological aging and health status are more relevant than simply setting a chronological age cut-off, particularly in HNC patients, who are generally more frail than other cancer patients [42]. In contrast, a recent study found that age is related to sarcopenic dysphagia [43]. However, this study employed a very different methodological setup and found a cut-off of 82 years, much higher than our threshold of 65 years.

Additionally, the analysis of muscle mass measurements revealed that gender is a significant factor in both SwMM and SMM. Males exhibited statistically significantly higher mean values across all muscle measurements compared to females. This underscores the need for sex-specific cut-off values when assessing sarcopenia, as using a uniform threshold could result in inaccurate diagnoses and management. This finding aligns with a previous study of Zwart et al., which supports that sex-specific SMI cut-off values are essential to diagnose low SMI [21]. Similarly, a study of Mori et al. found higher cut-off value for the geniohyoid muscle CSA men compared to women among healthy adults using ultrasonography [44].

Other studies have shown a relation between the swallowing muscles and SMI, based on muscle fiber composition. The tongue is mostly composed by type-1 fibers (S7), which are more susceptible to atrophy due to disuse rather than aging (S11). The association between the CSA of the tongue and SMI can be further explained by the fact that physical activity is related to SMI (S12), and higher tongue strength has been found in physically active older adults (S13).

Traditional and emerging techniques for assessing muscle mass have strengths and limitations. Dual-energy X-ray absorptiometry (DEXA) has been a standard method for measuring SMM but involves high cost and ionizing radiation [6]. Automated segmentation methods for body composition measurements using CT scans have facilitated their integration into clinical practice (S14, S15). However, abdominal CT scans are not part of the routine diagnostic procedures for HNC. Our study utilized CT scans of the head and neck area to measure muscle mass, based on the validated methodological framework of Swartz et al. to measure SMM at C3 level as alternative to abdominal scans. This approach is particularly relevant because head and neck CT scans are part of the standard diagnostic procedure in HNC, thus avoiding additional radiation exposure for muscle mass studies in a setting where minimizing radiation is a priority [12]. Furthermore, CSA measurements on CT and MRI scans at head and neck can be used interchangeably (S16), and there is no difference in SMM measurements between diagnostic neck CT scans and low-dose CT scans of the [^18^F]-FDG PET-CT scan (S17). Therefore, SMM can be reliably measured across different CT scans modalities when MRI is not available. However, measuring SwMM on MRI scans using our methodology still needs to be validated; therefore, we excluded patients with only MRI scans. Ultrasound is another widely used imaging technique for swallowing muscle mass. Studies has shown that in older population, the thickness of the masseter [45], and geniohyoid muscle [26] decreases with age. In older people with dysphagia, the CSA of masseter, genioglossus, geniohyoid and digastric muscles, also decreases with age [41]. Although ultrasound is a cost-effective and portable method for assessing swallowing muscle CSA, it is not everywhere part of standard diagnostic work-up settings [41].

This study has some limitations. The sample size, with less than half of eligible patients included, may limit the robustness of the findings. Although the study focused exclusively on HNC patients, the population was still heterogeneous, particularly in terms of tumor subsite and stage. Additionally, the use of surrogates such as SwMM, SMM at C3, SMM at L3, and SMI as proxies for whole-body muscle mass introduces potential variability in measurements of muscle mass composition in different individuals. This study also focused exclusively on muscle mass without assessing muscle strength or functional capacity, which would offer a more comprehensive evaluation of sarcopenia. As a consequence, these measurements alone may not fully reflect the complexities of muscle function and dysphagia in HNC patients. Although SwMM measurements were significant, they may be subject to potential variability in CT scan quality and interpretation. Lastly, the influence of nutritional status was not evaluated, which could play an essential role in SMM and SwMM.

Despite these limitations, our study has several strengths. First, the use of a validated methodological framework for SMM measurement and statistical analysis ensures the reliability and accuracy of the muscle mass data. Second, SwMM measurements followed a rigorous method assessing the swallowing muscle mass volume, with excellent inter-observer agreement. Third, patients were reselected from a previous study, where CSA measurements of the SMM at C3 level had excellent inter-observer and intra-observer agreement, providing reliable measurements. Finally, the short period between the diagnosis and SMM measurements (1.6 ± 2.4 weeks), minimized the influence of external and internal factors on muscle changes.

Future research should focus on exploring the relation between SwMM and functional swallowing assessment, as well as post-treatment swallowing outcomes, to better understand the direct impact of muscle mass on swallowing function. This relationship can provide insights into the pathophysiology of sarcopenic dysphagia, and a more objective development of targeted interventions. In addition, setting a gender-specific cut-off value for SwMM, would allow for an optimal stratification and identification of HNC population at higher risk of dysphagia. Furthermore, validating SwMM measurements through MRI could expand the sample size of this study, therefore enhancing the robustness of our findings and increasing the groups of patients who may benefit from these measurements.

## Conclusions

This study revealed significant correlations between SwMM and SMM at both the C3 and L3 vertebra levels, as well as with the SMI. However, the correlation between SwMM and SMI is not as strong as that of C3 for predicting SMI. Therefore, SwMM and C3 should not be considered interchangeable predictors of SMI. Although SwMM, measured by the TCM, and SMM, measured by SMI, are most likely two different entities, SwMM could be a very important predictor of sarcopenic dysphagia independent of SMM status. However, we did not evaluate sarcopenic dysphagia specifically in this study, thus necessitating future research on this topic to be able to confirm this hypothesis.

## Electronic supplementary material

Below is the link to the electronic supplementary material.

Supplementary Material 1
