# Supplementary material for: Unveiling the relation between swallowing muscle mass and skeletal muscle mass in head and neck cancer patients
Source: Eur Arch Otorhinolaryngol. 2025 Jan 25;282(6):3173–82. doi: 10.1007/s00405-025-09207-0 (PMC12122628; doi:10.1007/s00405-025-09207-0)
Supplement: Supplementary file 1 — Supplementary Material 1 [file 405_2025_9207_MOESM1_ESM.docx]

**Supplementary Material**

**Article Title:** Unveiling the relation between swallowing muscle mass and skeletal muscle mass in head and neck cancer patients

**Journal:** European Archives of Oto-Rhino-Laryngology

**Authors:** Javier Hurtado-Oliva, Lucy Núñez-Miranda, Aniek T. Zwart, Jeroen Vister, Boudewijn E.C. Plaat, Roel J.H.M. Steenbakkers, Anouk van der Hoorn, Inge Wegner, Gyorgy B. Halmos

**Corresponding author:**

Javier Hurtado-Oliva

Department of Otorhinolaryngology, Head and Neck Surgery, University Medical Center Groningen, University of Groningen, Groningen, The Netherlands

Departamento de Fonoaudiología, Facultad de Medicina, Universidad de Chile, Santiago, Chile

Email: j.a.hurtado.oliva@umcg.nl

**Supplementary material 1.** Supplemental references

S1. Sidorenkov G, Nagel J, Meijer C, Duker JJ, Groen HJM, Halmos GB, et al. The OncoLifeS data-biobank for oncology: a comprehensive repository of clinical data, biological samples, and the patient’s perspective. J Transl Med. 2019 Dec;17(1):374.

S2. Sobin LH, Gospodarowicz MK, Wittekind C. TNM Classification of Malignant Tumours. 7th ed. Oxford, UK: Wiley-Blackwell; 2009.

S3. Park A, Orlandini MF, Szor DJ, Junior UR, Tustumi F. The impact of sarcopenia on esophagectomy for cancer: a systematic review and meta-analysis. BMC Surg. 2023 Aug 17;23:240.

S4. Aro R, Mäkäräinen-Uhlbäck E, Ämmälä N, Rautio T, Ohtonen P, Saarnio J, et al. The impact of sarcopenia and myosteatosis on postoperative outcomes and 5-year survival in curatively operated colorectal cancer patients – A retrospective register study. Eur J Surg Oncol. 2020 Sep 1;46(9):1656–62.

S5. Kim MY, Kim SY, Shin HJ, Kweon KH, Park J, Kim NY. Effect of Sarcopenia on Pneumonia after Endoscopic Submucosal Resection in Patients Aged ≥65 Years: A Retrospective Study. Cancers. 2023 Sep 27;15(19):4753.

S6. Al-Azzawi Y, Albo B, Fasullo M, Coukos J, Watts GJ, Tai R, et al. Sarcopenia is associated with longer hospital stay and multiorgan dysfunction in alcoholic hepatitis. Eur J Gastroenterol Hepatol. 2020 Jun;32(6):733.

S7. Sanders I, Mu L, Amirali A, Su H, Sobotka S. The Human Tongue Slows Down to Speak: Muscle Fibers of the Human Tongue. Anat Rec. 2013;296(10):1615–27.

S11. Ciciliot S, Rossi AC, Dyar KA, Blaauw B, Schiaffino S. Muscle type and fiber type specificity in muscle wasting. Int J Biochem Cell Biol. 2013 Oct 1;45(10):2191–9.

S12. Takae R, Hatamoto Y, Yasukata J, Kose Y, Komiyama T, Ikenaga M, et al. Physical Activity and/or High Protein Intake Maintains Fat-Free Mass in Older People with Mild Disability; the Fukuoka Island City Study: A Cross-Sectional Study. Nutrients. 2019 Nov;11(11):2595.

S13. VanRavenhorst-Bell HA, Mefferd AS, Coufal KL, Scudder R, Patterson J. Tongue strength and endurance: Comparison in active and non-active young and older adults. Int J Speech Lang Pathol. 2017 Jan 2;19(1):77–86.

S14. Cespedes Feliciano EM, Popuri K, Cobzas D, Baracos VE, Beg MF, Khan AD, et al. Evaluation of automated computed tomography segmentation to assess body composition and mortality associations in cancer patients. J Cachexia Sarcopenia Muscle. 2020 Oct;11(5):1258–69.

S15. de Bree R, Meerkerk CDA, Halmos GB, Mäkitie AA, Homma A, Rodrigo JP, et al. Measurement of Sarcopenia in Head and Neck Cancer Patients and Its Association With Frailty. Front Oncol. 2022;12:884988.

S16. Zwart AT, Becker JN, Lamers MJ, Dierckx RAJO, de Bock GH, Halmos GB, et al. Skeletal muscle mass and sarcopenia can be determined with 1.5-T and 3-T neck MRI scans, in the event that no neck CT scan is performed. Eur Radiol. 2021 Jun;31(6):4053–62.

S17. Zwart AT, Cavalheiro VJ, Lamers MJ, Dierckx RAJO, de Bock GH, Halmos GB, et al. The validation of low-dose CT scans from the [18F]-FDG PET-CT scan to assess skeletal muscle mass in comparison with diagnostic neck CT scans. Eur J Nucl Med Mol Imaging. 2023;50(6):1735–42.

**Supplementary material 2.** Shapiro-Wilk normality test and Q-Q plots

| **Measurement** | **Statistic** | **df** | ***p*-value** |
| --- | --- | --- | --- |
| TCM | 0.994 | 157 | 0.811 |
| CSA at C3 | 0.977 | 157 | **0.010** |
| CSA at L3 | 0.987 | 157 | 0.146 |
| SMI | 0.992 | 157 | 0.521 |

*Legend: TCM = tongue complex muscles; CSA = cross sectional area; C3= third cervical vertebrae; L3 = third lumbar vertebrae; SMI = skeletal muscle index. Significant p-values values are indicated in bold.*

*
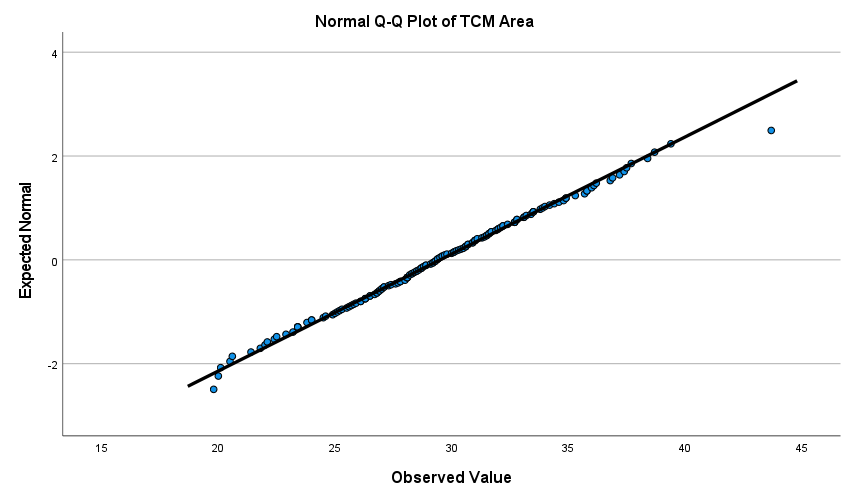
*

*
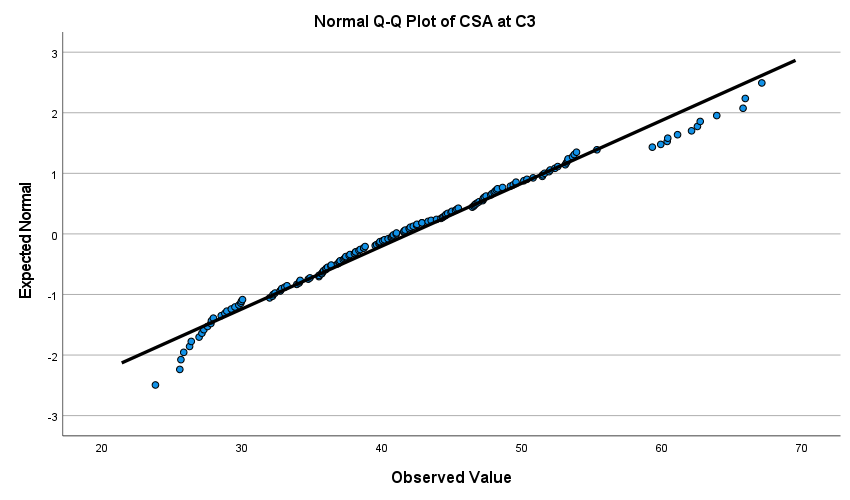
*

*
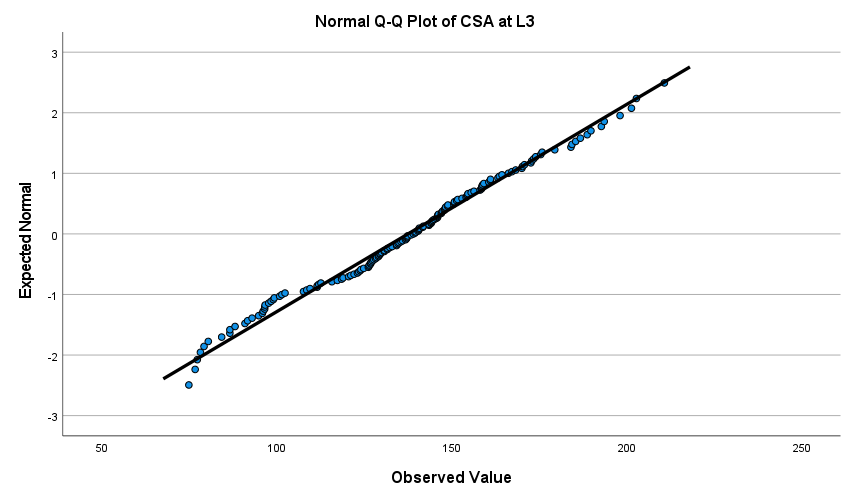
*

*
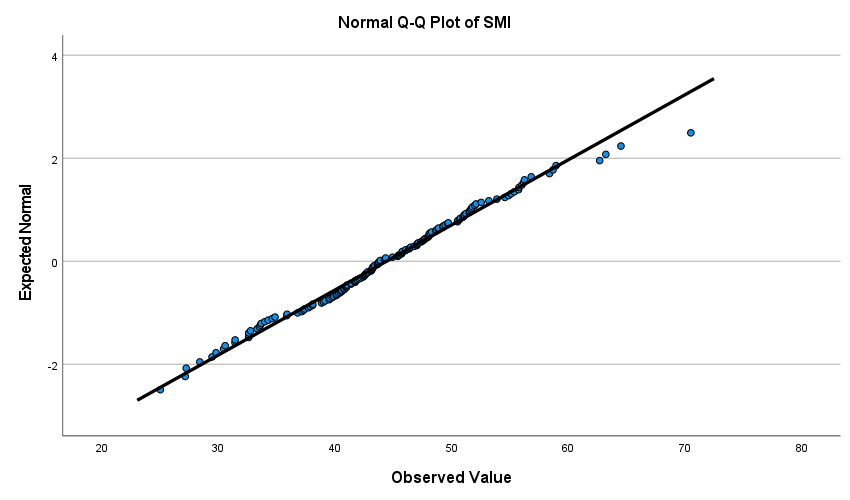
*
